# Exploring the Differential Diagnosis of Adrenal Adenoma in the Context of Situs Ambiguous: A Clinical Case Study

**DOI:** 10.3390/medicina60122010

**Published:** 2024-12-05

**Authors:** Pavel E. Stanchev, Mariya Dimitrova, Desislava Makakova, Boris Tilov

**Affiliations:** 1 Clinic of Endocrinology and Metabolic Diseases, St. George University Hospital, Medical University of Plovdiv, 4002 Plovdiv, Bulgaria; pavel.stanchev@mu-plovdiv.bg; 2 Department of Prosthetic Dentistry, Faculty of Dental Medicine, Medical University of Plovdiv, 4002 Plovdiv, Bulgaria; desislava.makakova@mu-plovdiv.bg; 3 Medical College, Medical University of Plovdiv, 4002 Plovdiv, Bulgaria; boris.tilov@mu-plovdiv.bg

**Keywords:** situs ambiguous, polysplenia, adrenal adenoma, congenital anomalies, endocrinology, adult situs inversus, anatomical variants

## Abstract

Situs anomalies, including situs inversus and situs ambiguous (SAMB), are rare congenital conditions typically noted in pediatric populations, with SAMB being particularly uncommon in adults. This case study addresses the incidental discovery of situs ambiguous with polysplenia in a 65-year-old man evaluated for suspected adrenal adenoma. The patient’s medical history included benign prostatic hyperplasia and tuberculous pleurisy. Methods included a thorough physical examination and laboratory tests, which showed normal cortisol levels and ACTH rhythm. Contrast-enhanced CT imaging revealed multiple spleens near the right adrenal region, altered liver positioning, a truncated pancreas, and a right-sided stomach, while the right adrenal gland was not visualized. Notably, the patient exhibited minimal symptoms despite these significant anatomical anomalies. The findings underscore the rarity of situs ambiguous in adults and its unexpected association with endocrine pathology. This case highlights the importance of comprehensive imaging and a multidisciplinary approach in managing patients with unusual anatomical presentations. It suggests that situs anomalies may be more prevalent in adult populations than previously recognized and emphasizes the need for increased clinical awareness and evaluation in similar cases.

## 1. Introduction

Situs solitus refers to the typical anatomical arrangement of the heart and abdominal organs. In this configuration, the cardiac apex, stomach, spleen, and aorta are located on the left side, while the liver and inferior vena cava are on the right [1]. The term “situs” specifically describes the positioning of internal organs and the heart concerning the body’s midline. Situs ambiguous is a rare congenital condition that can be divided into two main types: situs ambiguous with polysplenia (left isomerism) and situs ambiguous with asplenia (right isomerism) [2]. Polysplenia is characterized by multiple spleens, ranging from one to over ten, which may be located bilaterally or on the same side as the stomach. This arrangement reflects their development from the dorsal mesogastrium [3].

The clinical significance of situs ambiguous in adults is multifaceted and warrants further attention in the medical literature. Data on this condition in adult populations is relatively scarce, primarily because many individuals with situs ambiguous present with severe congenital anomalies, such as cardiac defects, immunodeficiencies, and gastrointestinal obstructions, which often limit their survival into adulthood [4]. Consequently, adult patients with situs ambiguous frequently exhibit mild or no symptoms, leading to a lower likelihood of diagnostic imaging unless conducted for unrelated health issues. This often results in most cases being discovered incidentally during examinations for other conditions, such as appendicitis or gallbladder disease. For instance, studies have shown that imaging findings related to situs ambiguous are often revealed during evaluations for acute abdominal issues, reinforcing the notion that awareness of this condition among healthcare providers is crucial [5].

Additionally, the presence of situs ambiguous can complicate the diagnosis of other conditions, including adrenal masses. Atypical anatomical arrangements can lead to the misinterpretation of imaging studies and pose challenges during surgical interventions, such as adrenalectomy, due to the potential for the misplacement of anatomical landmarks [6]. This highlights the necessity for clinicians to maintain a high index of suspicion for situs ambiguous in patients presenting with abdominal or endocrine symptoms, as early recognition can significantly impact management strategies.

Enhancing awareness and investigation into the manifestations of situs ambiguous in adult populations is essential, as further research could yield insights into outcomes, management strategies, and quality of life, potentially improving diagnostic and therapeutic approaches [7]. Recent studies have explored the link between situs ambiguous and various endocrine disorders, focusing on adrenal gland conditions. Adrenal adenomas, benign tumors originating from the adrenal cortex, are often identified incidentally during imaging for unrelated medical issues, including situs ambiguous [8]. While some adenomas secrete excess hormones, leading to conditions such as Cushing’s syndrome (excessive cortisol) or primary aldosteronism (elevated aldosterone), most are non-functional and asymptomatic [4].

Finding adrenal adenomas in situs ambiguous patients presents distinct challenges due to complex anatomical variations, requiring precise imaging and an understanding of unique anatomy [8,9]. Misinterpretation of imaging may lead to inappropriate management. Additionally, the link between adrenal adenomas and situs ambiguous suggests possible genetic predispositions [10,11]. Advancing the scientific understanding of these relationships highlights the need for heightened clinical awareness and multidisciplinary collaboration [12].

Abnormal organ positioning in situs ambiguous complicates adrenal lesion diagnosis and management [13]. However, advancements in imaging, like positron emission tomography (PET) and high-resolution magnetic resonance imaging (MRI), enhance the detection and characterization of adrenal adenomas, improving accuracy and treatment planning [14,15,16].

Research shows genetic mutations in situs ambiguous patients may raise susceptibility to endocrine disorders. Mutations in genes critical for left-right axis development, such as NR5A1 (SF-1), have been linked to adrenal dysgenesis and hormone imbalances [17,18]. Genetic screening may benefit those with situs ambiguous and endocrine abnormalities. Polysplenia and immunological deficiencies further heighten risks, including infections that affect the adrenal glands and cause adrenal insufficiency [19]. Over 80 genes contribute to normal L-R asymmetry in organ development, with a few identified in heterotaxy cases, like CRYPTIC, LEFTYA, and ZIC3. Further study is needed to clarify their roles in heterotaxy and related disorders [20,21].

In this case report, we present a patient with situs ambiguous, underscoring its rare occurrence as a potential factor in the differential diagnosis of adrenal adenoma, a frequently encountered endocrine disorder. This case report offers novel insights by exploring the rare association between situs ambiguous and adrenal adenomas, a connection not widely discussed in existing literature.

## 2. Case Presentation Section

A 65-year-old male was referred to the Endocrine Clinic for further evaluation after an ultrasound indicated a potential adenoma in the right adrenal gland. His medical history includes benign prostatic hyperplasia (BPH) and a previous diagnosis of tuberculous pleurisy.

### 2.1. Clinical Examination

Upon examination, the patient appeared to be in generally good health, with no visible skin discoloration. Auscultation of the lungs revealed clear bilateral breath sounds without wheezing. The cardiovascular assessment showed a regular heart rate of 82–84 beats per minute, with a blood pressure of 120/80 mmHg. Heart sounds were clear, and no cardiovascular abnormalities were detected. The abdominal examination revealed a soft, non-tender abdomen with normal peristalsis. Neither the liver nor spleen was palpable, and there was no tenderness on renal percussion. The extremities were free from swelling, with normal peripheral pulses and no signs of varicosities.

### 2.2. Laboratory Findings

The following laboratory tests were conducted as part of the patient’s evaluation. The results were within normal limits, with no indications of anemia or leukocytosis. The table below summarizes the key laboratory findings, including the normal reference ranges for each test. Additionally, stable blood pressure and normal potassium levels led to the decision to exclude measurements of renin and aldosterone, thereby ruling out mineralocorticoid hypertension (Table 1).

### 2.3. Abdominal CT Results

**Figure 1:**
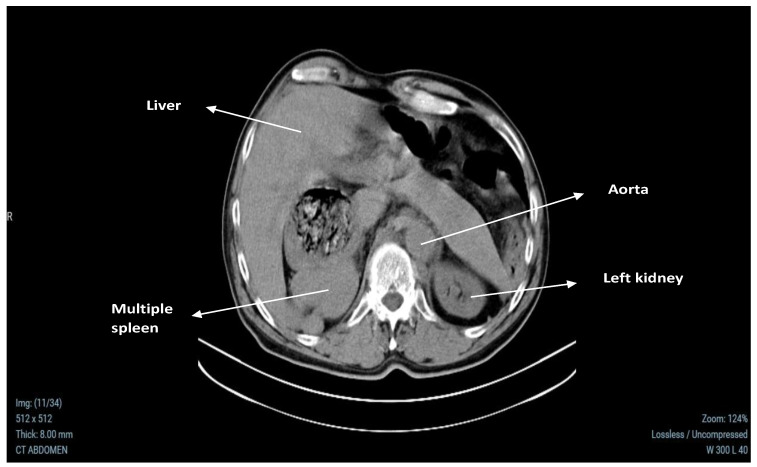
Native CT scan showing multiple spleens (accessory spleens) located in the area of the right adrenal gland. Accessory splenic tissue poses significant challenges in localizing and characterizing adrenal adenomas due to the potential mimicking of adrenal masses or distortion of typical anatomical landmarks.

**Figure 2:**
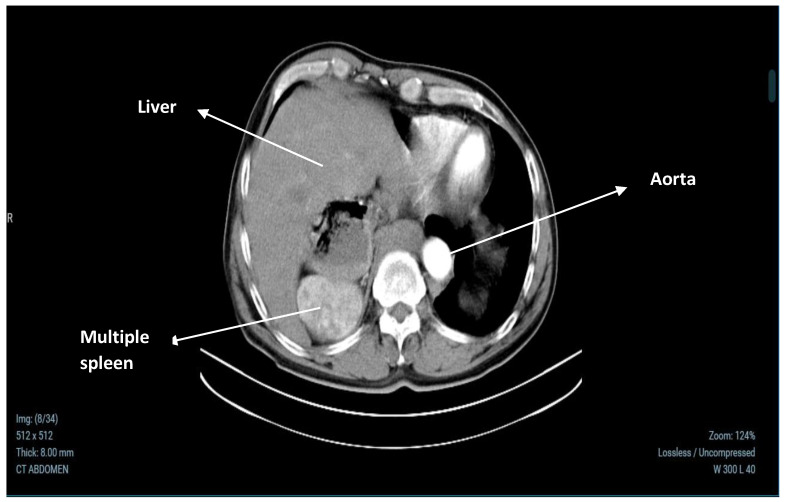
Contrast-enhanced CT scan demonstrating multiple accessory spleens situated in the region of the right adrenal gland. These accessory spleens can make it challenging to accurately localize and characterize adrenal lesions, as their contrast enhancement characteristics may closely resemble those of adrenal masses.

A contrast-enhanced abdominal CT scan revealed no abnormalities in the basal lung segments. The liver showed changes in its architecture, with separation of the left and caudate lobes, and three cysts were identified: one in the left lobe measuring 21.4 × 20 mm, another at 7.2 mm, and a third measuring 13.5 × 13.4 mm in segment 4A. There were no signs of intra- or extrahepatic cholestasis, and the portal vein was of normal size (18 mm). The gallbladder appeared normal in both structure and volume, filled with bile. The pancreas showed normal size and parenchyma, although its body and tail were not visualized, and no focal abnormalities were found. Both kidneys were of normal size and parenchyma, with the right kidney showing parenchymal cysts up to 15.1 mm in diameter, without evidence of drainage obstruction. The left adrenal gland appeared normal, but the right adrenal gland could not be visualized. The stomach was positioned entirely on the right side, with the esophagus terminating behind the right liver lobe at the cardiac orifice. No enlarged abdominal lymph nodes or free intra-abdominal fluid were present. In the region of the right adrenal gland, multiple splenic nodules were identified, the largest measuring 66 × 51 mm and showing homogeneous parenchymal echogenicity. These findings are documented in Figure 1 and Figure 2.

### 2.4. Psychological Assessment

The patient’s psychological assessment was conducted using a psychological interview by a clinical psychologist and the DASS-21 (Depression Anxiety Stress Scales-21), which provides a comprehensive overview of mental health status and is particularly suitable for patients with medical conditions, as in the present case. Despite the potential challenges of his medical condition, the patient demonstrated healthy adjustment and showed no signs of significant psychological distress.

### 2.5. Differential Diagnosis

The differential diagnosis is presented in the table below, illustrating the key differences between the patient’s clinical findings and the potential conditions that could explain the imaging and laboratory results (Table 2).

The table summarizes the distinctions between the patient’s clinical presentation and various potential differential diagnoses, emphasizing differences in symptoms, imaging characteristics, laboratory results, and overall clinical implications.

### 2.6. Conclusions and Plan

In summary, this patient presents with a stable clinical profile, with no evidence of adrenal hormonal dysfunction or other systemic abnormalities. However, the imaging findings suggest a need for further investigation of the suspected adrenal adenoma, particularly in the context of multiple splenic nodules in the right adrenal region and hepatic cysts. 

Further imaging or biopsy may be considered to better characterize the adrenal and splenic findings, as well as to assess the liver cysts. Routine follow-up and monitoring are recommended, particularly if new symptoms emerge.

## 3. Discussion

In the clinical case presented, we describe a patient with incomplete situs ambiguous who was evaluated for a suspected adenoma of the right adrenal gland. While the abdominal CT scan did not provide information regarding the position of the heart, a retrospective chest X-ray revealed no evidence of dextrocardia.

Our case analysis, supported by the existing literature, illustrates diagnosis and management complexities in situs ambiguous patients. Imaging techniques, particularly CT and MRI, differentiate benign adenomas from malignant tumors, with studies emphasizing critical radiological features for accuracy [22,23]. Functional adenomas often require surgical intervention, aligning with guidelines for improved outcomes [24,25]. Regular follow-up for hormonal imbalances or other issues is essential to prevent recurrence [26]. Multidisciplinary approaches tailored to complex anatomical variations optimize outcomes and minimize diagnostic or treatment delays (Table 3).

Situs inversus describes a rare congenital condition where internal organs are arranged in a mirror-image pattern compared to the standard configuration, situs solitus [32]. There are two main subtypes: situs inversus with dextrocardia (heart and viscera mirrored with the cardiac apex on the right) and situs inversus with levocardia (viscera mirrored but the cardiac apex remains on the left) [33,34,35] The dextrocardia form is more common, whereas situs inversus with levocardia is exceptionally rare and presents unique diagnostic and therapeutic challenges [36]. This distinction is crucial for healthcare providers, as these conditions can significantly impact surgical approaches and complication management.

Situs ambiguous involves a partial or complex arrangement of internal organs, typically associated with either polysplenia (multiple small spleens) or asplenia (absence of a spleen). This condition is rare in adults due to high mortality rates during early life, often linked to severe congenital heart defects [37,38,39]. Patients with milder cardiac issues may survive into adulthood [39,40]. In our case, we observed situs ambiguous with polysplenia, including multiple accessory spleens and complex organ malrotation. Such presentations are consistent with prior descriptions in the literature, although rare among adults [41,42,43].

Our case aligns with observations by Lama et al., who described a 59-year-old woman with situs ambiguous, featuring a persistent left superior vena cava and complex venous structures [44]. Such detailed imaging findings are vital for understanding organ arrangement variability and guiding clinical care. The significance of organ malrotation, as noted by Fulcher and Turner, highlights the diagnostic challenges posed by situs anomalies [45,46]. Accurate imaging is crucial, as atypical anatomy complicates diagnosis and treatment planning, often requiring tailored surgical approaches.

Similarly, Dres. Gonzalo Corral G. et al. analyzed four cases of situs ambiguous with polysplenia, noting consistent anatomical features such as multiple splenules and a shortened pancreas [47,48,49]. While three patients reported abdominal pain due to malrotation, one was asymptomatic, underscoring the variability in presentations. Our observations further emphasize the clinical importance of identifying anatomical variations for effective patient care.

Comparative literature reviews, including studies by Kim et al., show that advanced imaging technologies now allow for the incidental detection of situs ambiguous in asymptomatic adults [50]. This capability is crucial, as knowing a patient’s unique organ arrangement can guide treatment decisions and monitor potential complications like bowel obstruction or organ dysfunction. The expanding understanding of such cases has significantly enhanced patient care by informing more tailored management strategies.

The surgical management of conditions such as adrenal adenomas in patients with polysplenia also underscores the challenges associated with altered anatomy. Some studies note that complex vascular arrangements increase intraoperative risks and necessitate meticulous preoperative imaging [51,52]. Postoperative care must also account for immunological vulnerabilities in polysplenic individuals, as noted by Yilmaz et al., given their heightened infection risks [53,54]. Tailored approaches, including vaccination strategies and careful monitoring, are crucial to improving patient outcomes.

The comparison between our case and the broader literature highlights the need for heightened awareness of situs anomalies. Clinical management must consider both anatomical complexities and potential immune system implications. Understanding these variations can lead to improved diagnosis, surgical precision, and postoperative care, ultimately benefiting patients with complex congenital anomalies. As imaging technologies and diagnostic capabilities evolve, healthcare providers can develop increasingly effective strategies tailored to the unique needs of these patients.

Cases of situs ambiguous with polysplenia, such as ours, present a unique intersection of anatomical variability and clinical complexity. Comprehensive care approaches, informed by robust imaging and interdisciplinary collaboration, remain key to addressing the challenges posed by these rare anomalies. The consistent themes across comparative studies underscore the critical role of individualized diagnosis, surgical planning, and ongoing management in improving patient outcomes.

## 4. Conclusions

Situs anomalies, such as situs ambiguous, are often detected incidentally during imaging for unrelated concerns, like abdominal pain. In this case, the patient had polysplenia and a suspected right adrenal adenoma, which underscores the challenges of managing endocrine disorders in the presence of complex congenital abnormalities. While adrenal issues in situs ambiguous are rare, our review found no prior connections between situs ambiguous and adrenal adenomas or endocrine disorders. This case emphasizes the need for a collaborative approach among radiologists, endocrinologists, and surgeons, as well as the potential value of genetic testing to better understand and manage related complications.

## Figures and Tables

**Table 1:** Laboratory findings and normal reference ranges for the patient.

Laboratory Test	Result	Normal Range
Hemoglobin	15.2 g/dL	13.8–17.2 g/dL
White Blood Cells (WBC)	7126/µL	4000–11,000/µL
Blood Glucose	89 mg/dL	70–100 mg/dL
Creatinine	0.8 mg/dL	0.6–1.2 mg/dL
Erythrocyte Sedimentation Rate (ESR)	14 mm/h	0–22 mm/h
Electrolytes (Na^+^, K^+^, Cl^−^)	Na^+^: 139 mmol/L, K^+^: 4.3 mmol/L, Cl^−^: 101 mmol/L	Na^+^: 135–145 mmol/L, K^+^: 3.5–5.0 mmol/L, Cl^−^: 98–107 mmol/L
Liver Enzymes (ALT, AST, ALP, GGT)	ALT: 24 U/L, AST: 28 U/L, ALP: 98 U/L, GGT: 31 U/L	ALT: 7–56 U/L, AST: 10–40 U/L, ALP: 44–147 U/L, GGT: 9–48 U/L

**Table 2:** Comparative Analysis of Clinical Findings and Differential Diagnoses.

Aspect	Patient Case	Differential Diagnosis Summary
Patient Profile	A 65-year-old male with a history of benign prostatic hyperplasia and tuberculous pleurisy.	Patient profile is relevant for risk assessment. Age and comorbidities affect diagnostic considerations; older patients have a higher likelihood of malignancies or functional adrenal lesions.
Symptoms	Asymptomatic; referred for evaluation due to ultrasound findings.	- Pheochromocytoma: Typically presents with episodic hypertension, headaches, sweating, and palpitations, although may rarely be asymptomatic. - Adrenal Carcinoma: Symptoms often include abdominal discomfort, unexplained weight loss, or hormonal imbalances (e.g., Cushing’s syndrome). - Adrenal Adenomas: Usually incidental and asymptomatic; however, functioning adenomas may cause endocrine symptoms such as Cushing’s or Conn’s syndrome.
Adrenal Imaging Findings	Ultrasound indicated a potential adenoma; the right adrenal gland was not visualized on CT.	- Adrenal Adenoma: Often small (<4 cm), well-defined, with homogeneous features on imaging. Non-functional adenomas are more common. - Adrenal Carcinoma: Typically larger (>4 cm) and heterogeneous, with possible invasion into surrounding tissues. - Pheochromocytoma: Characterized by high vascularity; may exhibit enhancement on imaging and calcifications.
Laboratory Findings	Normal hormonal profile: preserved cortisol rhythm, normal ACTH, and 24 h urine-free cortisol. No signs of mineralocorticoid hypertension.	- Adrenal Adenoma: Hormonal overproduction, when present, is typically associated with cortisol (Cushing’s syndrome) or aldosterone (Conn’s syndrome). - Adrenal Carcinoma: Abnormal hormonal profiles include excessive cortisol, androgens, or aldosterone. - Pheochromocytoma: Diagnostic hallmark is elevated plasma or urinary metanephrines and catecholamines.
Other Imaging Findings	CT scan revealed liver cysts and multiple splenic nodules; normal kidney size with cysts.	- Metastatic Disease: Multiple lesions in the liver, spleen, or adrenal glands may indicate metastatic cancer. - Myelolipoma: Rare, benign adrenal mass often showing fatty components on imaging. - Splenic Lesions: Often benign (e.g., cysts, hemangiomas) but can indicate metastatic spread in systemic diseases.
Physical Examination	Good general health, no systemic signs; stable vital signs.	- Adrenal Carcinoma: Systemic illness signs such as unexplained weight loss, palpable mass, or systemic hypertension suggest malignancy. - Pheochromocytoma: May present with episodic or persistent hypertension; orthostatic hypotension may be noted.
Psychological Assessment	No significant mental health issues; demonstrates healthy adjustment levels.	Rarely directly addressed in differential diagnosis for adrenal disorders; however, chronic disease may affect psychological well-being.
Next Steps	Further imaging or biopsy may be considered to characterize findings.	- Adrenal Adenoma: Further hormonal assessment and imaging characterization are recommended. - Adrenal Carcinoma: Surgical biopsy may be necessary; staging imaging is crucial. - Pheochromocytoma: Confirmation with biochemical testing followed by functional imaging (e.g., MIBG scan).

**Table 3:** Comparison of adrenal adenoma case studies with focus on diagnosis, treatment, and follow-up: a review of published literature.

Case №	Age	Gender	Country	Symptoms	Diagnosis	Treatment	Follow-Up	References
1.	55	Male	USA	Incidental finding on CT	Non-functional adrenal adenoma	Observation	6 months, stable	Tariq et al., 2012 [27]
2.	62	Female	Japan	Flank pain, hypertension	Adrenal adenoma with metastasis	Surgical excision, 1 week after diagnosing	12 months (no recurrence)	Choi et al., 2013 [24]
3.	48	Male	South Korea	Fatigue, weight loss	Adrenal adenoma (metastasis from RCC)	Adrenalectomy, 3 weeks after diagnosing	9 months (stable)	Moosavi et al., 2016 [28]
4.	39	Female	UK	Incidental mass on MRI	Benign adrenal adenoma	Monitoring	18 months (no growth)	Lam and Lo, 2002 [26]
5.	60	Male	Italy	Palpitations, dizziness	Functional adrenal adenoma	Laparoscopic adrenalectomy, 10 days after diagnosing	12 months (normal cortisol levels)	Boland et al., 2009 [22]
6.	45	Female	Canada	Nausea, fatigue, incidental mass	Non-functional adrenal adenoma	Monitoring	24 months (stable mass size)	Sasaguri et al., 2016 [25]
7.	52	Male	Australia	Weakness, hypertension	Functional adrenal adenoma (Conn’s syndrome)	Surgical removal, 2 weeks after diagnosing	10 months (improved BP)	Schieda et al., 2017 [23]
8.	65	Female	USA	Abdominal pain, incidental mass	Adrenal adenoma	Adrenalectomy, 3 weeks after diagnosing	6 months (no recurrence)	Tsushima et al., 2009 [29]
9.	47	Male	UK	Weight gain, hypertension	Functional adrenal adenoma	Adrenalectomy, 3 weeks after diagnosing	14 months (normalized BP)	Blake et al., 2010 [30]
10.	58	Female	USA	Incidental mass on CT	Benign adrenal adenoma	Observation	12 months (stable)	Korobkin et al., 1996 [31]

## Data Availability

The original contributions presented in this study are included in the article. Further inquiries can be directed to the corresponding author.
